# Analysis of Circadian Rhythms in the Basal Filamentous Ascomycete *Pyronema confluens*

**DOI:** 10.1534/g3.115.020461

**Published:** 2015-08-07

**Authors:** Stefanie Traeger, Minou Nowrousian

**Affiliations:** Lehrstuhl für Allgemeine und Molekulare Botanik, Ruhr-Universität Bochum, 44780 Bochum, Germany

**Keywords:** circadian clock, *frequency*, clock-controlled genes, light-entrainment, *Pyronema confluens*

## Abstract

Many organisms use circadian clocks to adapt to daily changes in the environment. Major insights into the molecular mechanisms of circadian oscillators have been gained through studies of the model organism *Neurospora crassa*; however, little is known about molecular components of circadian clocks in other fungi. An important part of the *N. crassa* circadian clock is the *frequency* (*frq*) gene, homologs of which can be found in Sordariomycetes, Dothideomycetes, and Leotiomycetes, but not Eurotiomycetes. Recently, we identified a *frq* homolog in *Pyronema confluens*, a member of the early-diverging Pezizomycete lineage of filamentous ascomycetes. The *P. confluens* FRQ shares many conserved domains with the *N. crassa* FRQ. However, there is no known morphological phenotype showing overt circadian rhythmicity in *P. confluens*. To investigate whether a molecular clock is present, we analyzed *frq* transcription in constant darkness, and found circadian oscillation of *frq* with a peak in the subjective morning. This rhythm was temperature compensated. To identify additional clock-controlled genes, we performed RNA sequencing of two time points (subjective morning and evening). Circadian expression of two morning-specific genes was verified by reverse transcription quantitative polymerase chain reaction (RT-qPCR) over a full time course, whereas expression of two putative morning-specific and five putative evening-specific genes could not be verified as circadian. *frq* expression was synchronized, but not entrained by light. In summary, we have found evidence for two of the three main properties of circadian rhythms (free-running rhythm, temperature compensation) in *P. confluens*, suggesting that a circadian clock with rhythmically expressed *frq* is present in this basal filamentous ascomycete.

Adaptation to daily changes in the environment, *e.g.*, fluctuating temperatures and light levels, is a central feature in the life of most organisms. Circadian clocks aid with these adaptations by enabling cells and organisms to anticipate such changes instead of simply responding to them (Dunlap and Loros 2006; Fuller *et al.* 2014). Circadian systems can be found in prokaryotes and eukaryotes, and the molecular mechanisms generating these rhythms have been studied in model organisms as varied as the cyanobacterium *Synechococcus elongatus*, the plant *Arabidopsis thaliana*, the fungus *Neurospora crassa*, the fly *Drosophila melanogaster*, and mammals, *e.g.*, mice (Bell-Pedersen *et al.* 2005; Salomé and McClung 2004; Sancar and Brunner 2014). Major contributions toward elucidating clock mechanisms have come from studies on circadian rhythms in the filamentous ascomycete *N. crassa* (Dunlap 2006; Heintzen and Liu 2007). In several other fungi, circadian rhythms have been described for different morphologic or metabolic outputs; however, little is known about molecular components of circadian oscillators in these species (Greene *et al.* 2003; Hurley *et al.* 2015; Loros and Dunlap 2001; Oliveira *et al.* 2015).

Circadian rhythms can be defined by three characteristic features, namely a period length of about 24 hr, the ability to be reset or entrained by periodic light or temperature cues, and the ability to run stably within the physiological range of changes in nutrients or temperature (temperature compensation) (Bell-Pedersen *et al.* 2005; Fuller *et al.* 2014). In *N. crassa*, an easily observable phenotypic output of the circadian clock is the rhythmic conidiation; and the molecular machinery driving circadian output is the FWO or FRQ/WCC (white collar complex) oscillator, which is named for three of its essential components, the *frequency* (*frq*), *white collar-1* (*wc-1*), and *white collar-2* (*wc-2*) genes (Fuller *et al.* 2014). The FWO organizes a stable circadian rhythm via a transcriptional/translational feedback loop that requires two main protein complexes. One is the WCC consisting of the photoreceptor WC-1 and the WC-2 protein, and the second comprises FRQ and FRQ-interacting helicase (FRH) (Fuller *et al.* 2014). The WCC acts as the positive arm in the oscillator loop by binding the *frq* promoter and activating *frq* transcription (Froehlich *et al.* 2002, 2003). FRQ protein is made and interacts with FRH, and the resulting complex acts as the negative arm of the oscillator by binding to the WCC, leading to phosphorylation and inactivation of this complex (Cheng *et al.* 2005; He *et al.* 2006; Schafmeier *et al.* 2005). This, in turn, leads to reduced *frq* transcription and subsequently to a decrease in FRQ protein levels. Once FRQ/FRH-mediated inhibition of the WCC is reduced, the WCC resumes its activity and the cycle starts again.

Besides the FWO, other oscillators in *N. crassa* have been collectively described as FLOs or *frq*-less-oscillators (De Paula *et al.* 2006; Dragovic *et al.* 2002; Lakin-Thomas *et al.* 2011; Nsa *et al.* 2015). Molecular components of the FLOs that are not also part of the FWO were largely unknown until the recent characterization of an oscillator that does not require *frq* but the cryptochrome-encoding *cry* gene and was therefore named *cry*-dependent oscillator (Nsa *et al.* 2015). However, most FLOs, including the *cry*-dependent oscillator, lack some of the defining properties of circadian rhythms, giving rise to the hypothesis that the FWO is the major oscillator in *N. crassa* with other oscillators being ancillary or active only under certain physiological conditions (Dunlap and Loros 2004).

The *frq* gene as a core component of the FWO has been studied intensively for more than 20 yr, and besides the aforementioned regulatory events, has been found to be regulated at every conceivable level of expression to fine-tune the *N. crassa* clock under a wide range of environmental conditions (Cha *et al.* 2015; Fuller *et al.* 2014). Interestingly though, FRQ is the least conserved among the known oscillator proteins in *N. crassa*. Whereas FRH is a conserved eukaryotic protein, and WC-1 and WC-2 are conserved widely in fungi from zygomycetes to ascomycetes and basidiomycetes, until recently FRQ homologs were thought to be restricted to the Sordariomycetes, Leotiomycetes, and Dothideomycetes groups of filamentous ascomycetes (Cheng *et al.* 2005; Dunlap and Loros 2006; Salichos and Rokas 2010). The recent characterization of a *frq*-dependent circadian clock in the Leotiomycete *Botrytis cinerea* indicates that *frq* has a function in circadian oscillators in fungal groups that evolved in parallel to *N. crassa* (Hevia *et al.* 2015). Sequencing the genome of the Pezizomycete *Pyronema confluens* revealed a *frq* homolog (*PCON_09365*), indicating that *frq* was present in the ancestor of filamentous ascomycetes, because the Pezizomycetes represent an early-diverging lineage that evolved in parallel to the Sordariomycetes, Leotiomycetes, Dothideomycetes, and Eurotiomycetes (Traeger *et al.* 2013). This also implies that *frq* was probably lost in the ancestor of the Eurotiomycetes, which is the only lineage of filamentous ascomycetes where no *frq* homolog has been found to date (Salichos and Rokas 2010).

The *P. confluens frq* is light induced with blue light being the activating part of the visible spectrum, similar to its *N. crassa* counterpart (Crosthwaite *et al.* 1995; Froehlich *et al.* 2002; Traeger *et al.* 2013). Furthermore, analysis of the *P. confluens* genome as well as expression analyses revealed that homologs of other clock components, namely WC-1, WC-2, and the downstream transcription factor SUB-1, are present and expressed in *P. confluens* (Traeger *et al.* 2013). Therefore, we wondered whether a circadian clock was present in this fungus and whether other properties of the *P. confluens frq* besides light induction were also similar to *N. crassa*, namely a circadian regulation of transcript levels.

Here, we present evidence that *P. confluens frq* transcript levels are rhythmic and temperature-compensated. Furthermore, we performed RNA sequencing (RNA-seq) for two different time points during the circadian cycle and identified several putative clock-controlled genes (*ccgs*). Circadian expression under free-running conditions could be verified for two morning-specific genes by reverse transcription quantitative polymerase chain reaction (RT-qPCR). Our data suggest that a circadian clock with a rhythmically expressed *frq* gene is active in the basal filamentous ascomycete *P. confluens*.

## Materials and Methods

### Strains and culture conditions

*P. confluens* CBS100304 was grown on minimal medium as described previously (Nowrousian and Kück 2006; Traeger *et al.* 2013). For analysis of circadian rhythms, *P. confluens* was grown in shaken cultures in minimal medium (75 mL in 100-mL Erlenmeyer flask) at 25° or 30° that were inoculated with mycelial plugs from 3-d cultures in liquid medium as described (Nowrousian and Kück 2006). Cultures were entrained for at least one day in constant white light (LL), shifted to dark (DD) in 4-hr intervals over 2 d (Supporting Information, Figure S1), and harvested under red light as described (Nowrousian and Kück 2006). Previous analyses had shown that *P. confluens* is not sensitive to red light (Nowrousian and Kück 2006; Traeger *et al.* 2013). For light entrainment analysis, 100-mL cultures inoculated with mycelial plugs were kept at 25° in LL for at least 4 hr and were subsequently subjected to dark/light cycles (9 hr/9 hr, 12 hr/12 hr, or 14 hr/14 hr) and harvested under red light at defined intervals before and after “lights on” on the fourth day (Figure S2). For analyses of free-running rhythms as well as entrainment, cultures at the time of harvest had approximately the same age (72−80 hr). Under the shaking conditions used, mycelial plugs reached a stationary phase (cease of mycelial growth) after about 2.5 d, therefore cultures were in stationary phase at the time of harvest.

### RNA extraction and RT-qPCR analysis

*P. confluens* RNA was prepared with the RNeasy lipid tissue mini kit (QIAGEN, Hilden, Germany) with modifications as described (Nowrousian and Kück 2006). Reverse-transcription and RT-qPCR were performed as described previously (Gesing *et al.* 2013; Schindler and Nowrousian 2014). Oligonucleotide primers for RT-qPCR are given in Table S1. Statistical analysis of rhythmicity (period length 20−28 hr) was performed with JTK_CYCLE (Hughes *et al.* 2010) in the R computing environment (version 3.1.1) using the normalized expression values (greatest value set to 100%) for all replicates at 25° and (if performed) at 30° for each gene for analysis as described in the JTK_CYCLE manual, with genes with *P*-values ≤ 0.01 regarded as rhythmic.

### RNA-seq analysis

For Illumina/Solexa RNA-Seq analysis, RNA of three independent biological replicates of the subjective morning (DD36) and subjective evening (DD24) was sequenced at GATC Biotech (Konstanz, Germany). Raw data were analyzed and trimmed as described previously and mapped to the reference genome v01_2 of *P. confluens* (Teichert *et al.* 2012; Traeger *et al.* 2013). For quantitative analysis of gene expression, DESeq and LOX were used to calculate expression ratios (morning *vs.* evening) for all predicted genes (Anders and Huber 2010; Zhang *et al.* 2010).

### Phylogenetic analysis

Multiple alignments were created in CLUSTALX (Thompson *et al.* 1997), manually adjusted, and visualized with Jalview (Waterhouse *et al.* 2009), and the same alignment was used for analysis by neighbor joining or maximum parsimony. Phylogenetic analyses were made with PAUP version 4.0b10 for Windows (D.L. Swofford, distributed by Sinauer Associates, copyright 2001 Smithsonian Institution). Neighbor joining and maximum parsimony analyses were performed as described using 1000 bootstrap replicates (Hall 2004). Consensus trees were graphically displayed with Dendroscope (Huson *et al.* 2007).

### Data availability

The RNA-seq reads and derived expression ratios generated in this study were submitted to the GEO database (accession number GSE61263). The Supporting Information contains Figure S1, Figure S2, Figure S3, and Figure S4, as well as Table S1 and Table S3. Table S2 is presented in a separate Excel file. Figure S1, growth regime for analysis of free-running rhythms in *P. confluens*. Figure S2, growth regime for analysis of light entrainment in *P. confluens*. Figure S3, phylogenetic analysis of FRQ homologs from different ascomycetes. Figure S4, corrected genomic sequence of the *S. macrospora frq* homolog (*SMAC_03705*). Table S1, oblignucleotides used in the study. Table S2, analysis of differential gene expression at two different time points (DD24, DD36) in *P. confluens* by RNQ-seq. Table S3, short term light induction of putative clock-controlled genes.

## Results

### *P. confluens* FRQ comprises conserved domains, and *frq* expression is rhythmic and temperature-compensated

Circadian clocks are characterized by three characteristics: (1) They sustain a near-24-hr rhythm under constant conditions; (2) the rhythm is stable under a wide range of conditions, *e.g.*, at different temperatures; and (3) the rhythm can be entrained, *i.e.*, it can adjust to environmental cues like day−night cycles (Dunlap *et al.* 2007). In *N. crassa*, an easily visible phenotypic output from the clock is the rhythmic banding of conidiation on race tubes in constant darkness (DD); *P. confluens*, however, does not produce any conidia. Fruiting body formation in *P. confluens* is strictly light-dependent (Claussen 1912; Gwynne-Vaughan and Williamson 1931; Traeger *et al.* 2013), and therefore cannot be observed in DD; and in our analyses, fruiting body formation was not rhythmic in the light (data not shown). Thus, at present there is no morphologic phenotype that shows circadian rhythmicity in *P. confluens*; however, the presence of an *frq* gene opens the possibility that this gene might be associated with circadian rhythmicity and that a circadian clock might be active at the molecular level in this fungus.

**Figure 1:**
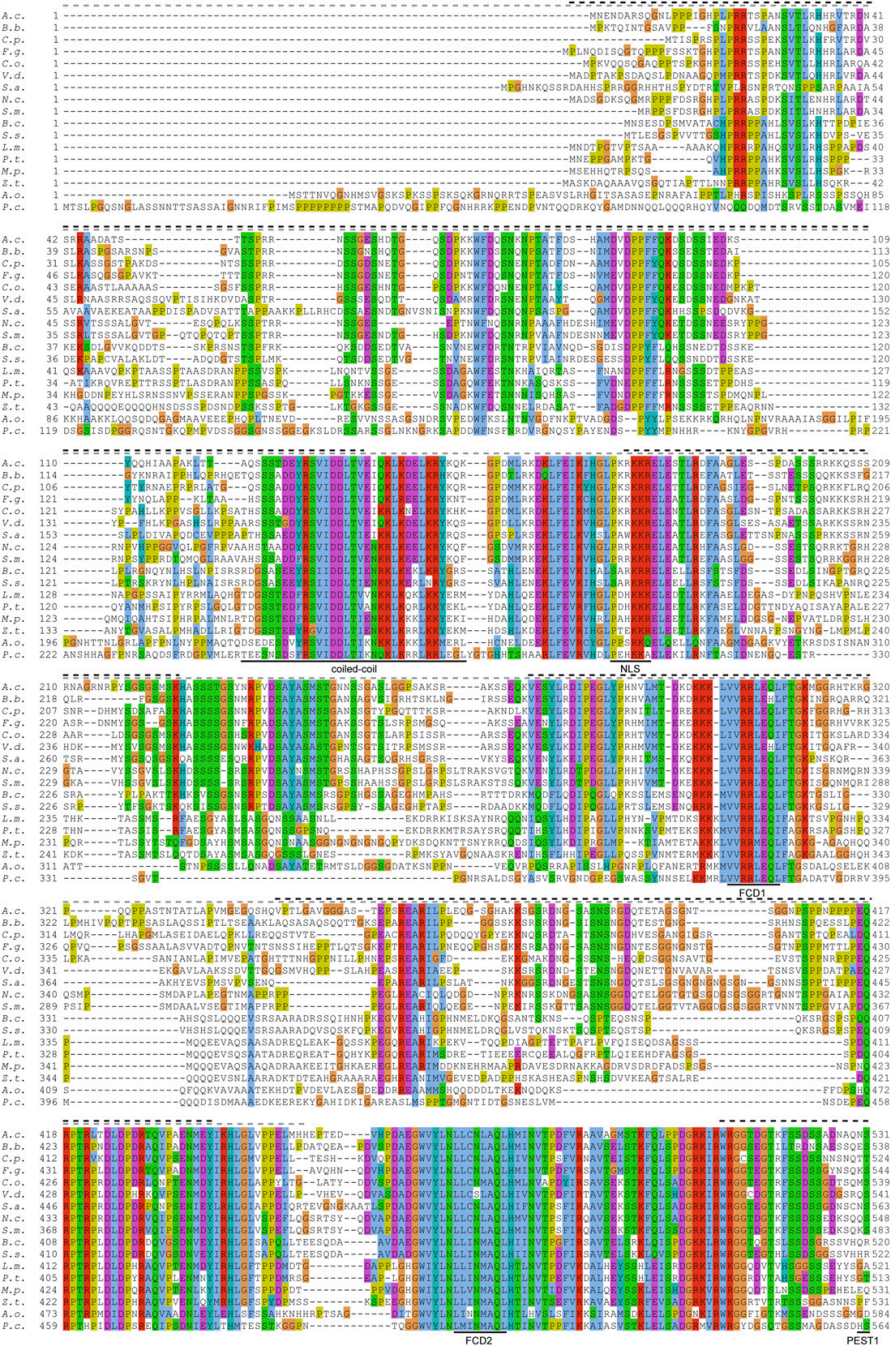

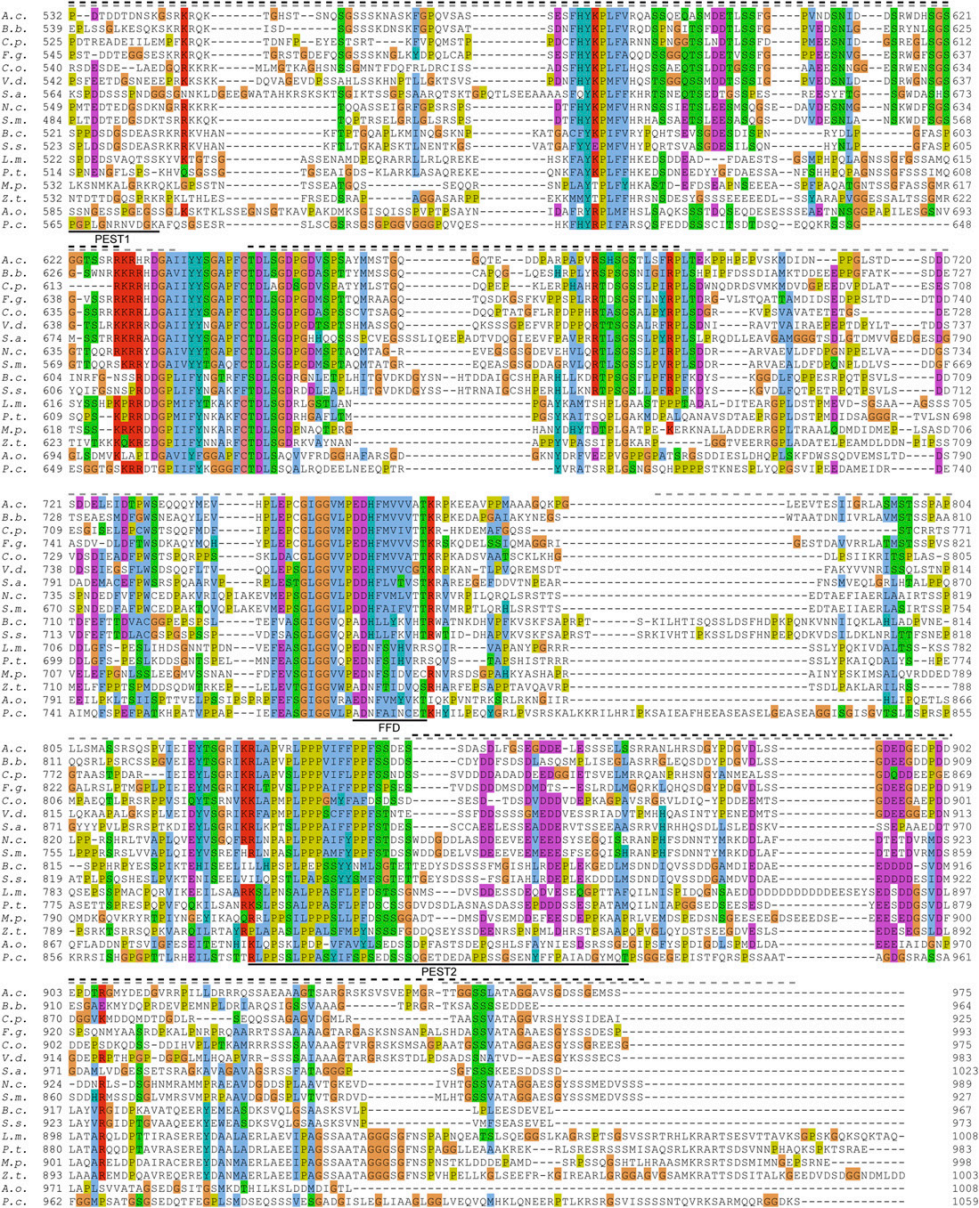
Multiple alignment of FRQ homologs from different ascomycetes. Protein domains that were characterized in the *N. crassa* FRQ are indicated below the alignment: Coiled-coil domain (Cheng *et al.* 2001); FCD, FRQ-CK1a interaction domain (He *et al.* 2006; Querfurth *et al.* 2011); FFD, FRQ/FRH interaction domain (Guo *et al.* 2010); NLS, nuclear localization signal (Luo *et al.* 1998); PEST domains (Merrow and Dunlap 1994). Intrinsically unstructured regions were predicted with IUPred (Dosztányi *et al.* 2005) for the *N. crassa* and *P. confluens* FRQ proteins, indicated by dashed black and gray lines, respectively, above the sequence. The following sequences were used: *A.c. Acremonium chrysogenum* gb|KFH48196.1; *A.o. Arthrobotrys oligospora* gb|EGX51094.1; *B.b. Beauveria bassiana* ref|XP_008594847.1; *B.c. Botrytis cinerea* ref|XP_001547609.1; *C.o. Colletotrichum orbiculare* gb|ENH76664.1; *C.p. Claviceps purpurea* emb|CCE27108.1; *F.g. Fusarium graminearum* gb|ESU12098.1; *L.m. Leptosphaeria maculans* ref|XP_003845311.1; *M.p. Macrophomina phaseolina* gb|EKG17747.1; *N.c. Neurospora crassa* gb|AAA57121.1; *P.t. Pyrenophora tritici-repentis* ref|XP_001941961.1; *P.c. Pyronema confluens* PCON_09365; *S.a. Scedosporium apiospermum* gb|KEZ43134.1; *S.m. Sordaria macrospora* ref|XP_003351398.1 was manually reannotated after correction of errors in the genome sequence (Figure S4); *S.s. Sclerotinia sclerotiorum* SS1G_11844.3; *V.d. Verticillium dahliae* gb|EGY17172.1; *Z.t. Zymoseptoria tritici* ref|XP_003851799.1. The alignment was visualized in Jalview using the ClustalX color scheme (Thompson *et al.* 1997; Waterhouse *et al.* 2009).

The *P. confluens frq* gene was the first to be discovered in the early-diverging Pezizomycete lineage, pushing the evolutionary origin of *frq* back to (at least) the last common ancestor of filamentous ascomycetes (Traeger *et al.* 2013). This is also supported by the presence of a *frq* homolog in *Arthrobotrys oligospora*, a species that belongs to the Orbiliomycetes, a sister group of the Pezizomycetes (Traeger *et al.* 2013; Yang *et al.* 2011) (Figure 1, Figure S3). The FRQ homologs from these two species are moderately conserved across the full length of the predicted proteins with several highly conserved interspersed regions, and cluster together in a phylogenetic analysis of FRQ proteins from different ascomycete groups as expected (Figure 1, Figure S3).

The *N. crassa* FRQ protein comprises several protein domains that were shown to be involved in clock functions. These domains show varying degrees of conservation in *P. confluens* (Figure 1). Of the two PEST domains, both of which are phosphorylated and necessary for rhythmicity in *N. crassa* (Görl *et al.* 2001; Merrow and Dunlap 1994; Schafmeier *et al.* 2006), the second shows stronger conservation than the first (Figure 1). In *N. crassa*, the two PEST domains have distinct functions, with PEST1 involved in determining period length, and PEST2 involved in cytoplasmic accumulation of the WCC (Görl *et al.* 2001; Schafmeier *et al.* 2006). The greater conservation of PEST2 could indicate a more conserved function for this domain, or alternatively the function of PEST1 might be less dependent on actual sequence and rather on the ability to be phosphorylated. A coiled-coil domain in the *N. crassa* FRQ was shown to be essential for rhythmic conidiation and for the interaction of FRQ with itself (Cheng *et al.* 2001). The coiled-coil domain is conserved in most FRQ homologs, including two leucine residues (positions 165 and 169) that are important for FRQ-FRQ interaction in *N. crassa* (Cheng *et al.* 2001). Two FRQ-CK1a interaction domains (FCD1 and FCD2) are strongly conserved in *P. confluens* (Figure 1). In *N. crassa*, the domains are important for the interaction of casein kinase 1a with FRQ and its subsequent phosphorylation (He *et al.* 2006; Querfurth *et al.* 2011). In contrast, the FRQ/FRH interaction domain (FFD) is only moderately conserved in *P. confluens*. In *N. crassa*, only the FRQ−FRH complex can interact with the WCC and sustain stable rhythmicity (Guo *et al.* 2010). Whether the partially conserved FFD might support interaction with the *P. confluens* FRH homolog (PCON_11360) remains to be elucidated. The nuclear localization signal present in the *N. crassa* FRQ (Luo *et al.* 1998) is not conserved in *P. confluens*; however, PSORT (Nakai and Horton 1999) predicts the *P. confluens* FRQ as a nuclear protein with several putative nuclear localization signals located elsewhere in the protein (data not shown). Apart from the presence of several functional domains, it was shown that the *N. crassa* FRQ is an intrinsically disordered protein that contains large regions of low structural complexity (Hurley *et al.* 2013; Querfurth *et al.* 2011). Using IUPred (Dosztányi *et al.* 2005), a similar tendency was observed in the *P. confluens* FRQ (Figure 1). Overall, the conservation of many functionally important domains combined with large regions of low structural complexity indicates that major features of FRQ that were identified in *N. crassa* are conserved in *P. confluens* and other fungal species and therefore might have been already present in the last common ancestor of filamentous ascomycetes. In addition, there are several regions conserved across FRQ homologs from filamentous ascomycetes for which no molecular roles have been assigned yet (Figure 1). These might be potential regions of interest for future functional studies.

**Figure 2:**
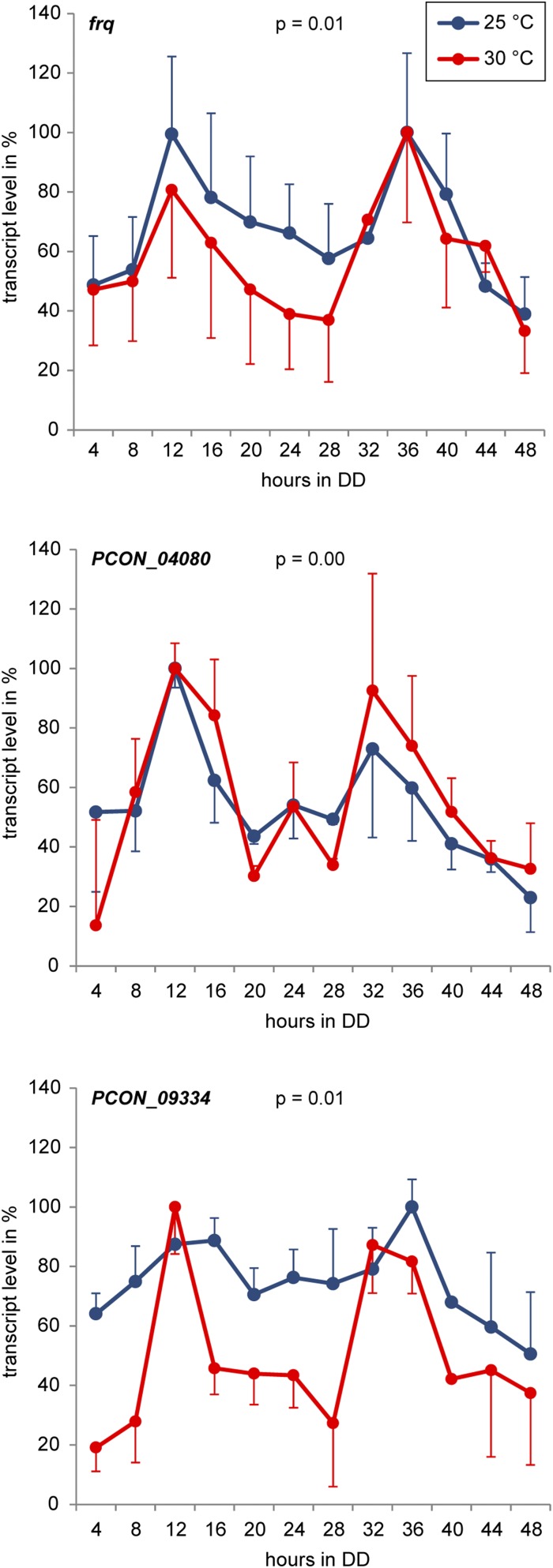
Expression of *P. confluens frq* and two putative *ccgs* is rhythmic under free-running conditions. Transcript levels were determined by RT-qPCR after the indicated times in constant darkness (DD). The growth regime is shown in Figure S1. Means and standard errors from three independent replicates at 25° and two independent replicates at 30°, respectively, are shown. The greatest value in each time course was set to 100%. Standard errors are shown in one direction only (up or down) for better visualization. Statistical analysis of circadian expression was performed using JTK_CYCLE (Hughes *et al.* 2010), *P*-values for an analysis including all replicates at both temperatures are given after the corresponding gene names.

In *N. crassa*, *frq* transcript levels are rhythmic in DD, and this rhythm is temperature-compensated within the physiological temperature range for rhythmicity (Aronson *et al.* 1994; Liu *et al.* 1998). To check whether expression of the *P. confluens frq* is also rhythmic and temperature-compensated, we analyzed transcript levels by RT-qPCR during a 48-hr time course in DD at two different temperatures (Figure 2). *frq* expression peaked in the subjective morning, *i.e.*, ∼12 and ∼36 hr after transfer to DD, with a ∼24-hr rhythm that was sustained at 25 and 30°, indicating temperature-compensation. Statistical analysis with JTK_CYCLE (Hughes *et al.* 2010) confirmed rhythmicity, despite high variability of individual time courses leading to large error bars in Figure 2 (see *Discussion*). Thus, *frq* in *P. confluens* shows properties of a clock-controlled gene (*ccg*). Whether it is part of a circadian oscillator machinery cannot be concluded from these data.

### Identification of ccgs by RNA-seq

**Figure 3:**
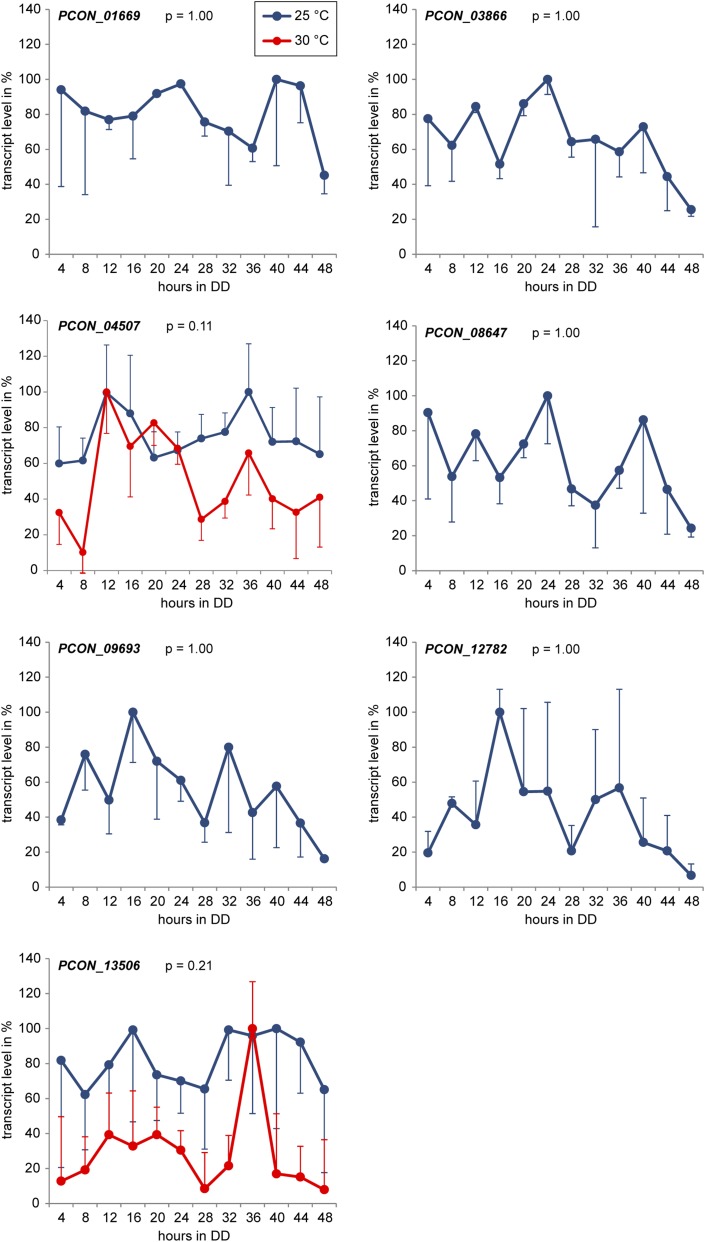
Expression analysis of seven putative *ccgs* that are not rhythmic under free-running conditions. Transcript levels were determined by RT-qPCR after the indicated times in constant darkness (DD). Means and standard errors from three independent replicates at 25° and two independent replicates at 30° (*PCON_04507* and *PCON_13506*) or from two independent replicates at 25° (all others) are shown. The greatest value in each time course was set to 100%. Standard errors are shown in one direction only (up or down) for better visualization. Statistical analysis of circadian expression was performed using JTK_CYCLE (Hughes *et al.* 2010), *P*-values for an analysis including all replicates (at both temperatures if applicable) are given after the corresponding gene names.

To search for *ccgs* besides *frq*, we performed RNA-seq experiments with samples from two different time points during the circadian day representing the subjective evening and subjective morning. RNA was extracted from samples 24 hr (subjective evening) and 36 hr (subjective morning) after transfer to DD. RNA-seq was performed on three independent biological replicates per time point (Table 1). Differential gene expression analysis to identify genes that were up-regulated during the subjective morning or subjective evening was performed with DESeq and LOX (Anders and Huber 2010; Zhang *et al.* 2010). Expression data for all *P. confluens* genes as well as for the 80 most significantly regulated genes are presented in Table S2. Interestingly, of the 80 most significantly regulated genes, only eight were up-regulated in DD36 *vs.* DD24 and thus putative morning-specific *ccgs*, whereas the others were down-regulated and thus putative evening-specific *ccgs* (Table S2). To test whether putative *ccgs* identified by RNA-seq showed truly circadian expression patterns, four putative morning-specific and five evening-specific genes chosen from the 80 most significantly regulated genes were analyzed over a 48-hr time course by RT-qPCR (Figure 2, Figure 3, and Table 2). However, only expression of two of the morning-specific genes could be verified as circadian at 25° and 30°, whereas the five evening-specific genes were arrhythmic when tested at 25°. The two remaining morning-specific genes were also arrhythmic, but with better *P*-values in a JTK_CYCLE test for circadian rhythmicity (Hughes *et al.* 2010), suggesting that they might be under weak clock control (Figure 3). Overall, two morning-specific genes were verified as *ccgs* with rhythmic and temperature-compensated expression.

**Table 1:** Overview of RNA-seq experiments for two time points (DD24 and DD36)

Sample Name	Sample	No. of Reads	No. of Trimmed Reads	No. of Mapped Reads	% of Reads That Map
Pcon13	DD24_a	18,998,703	18,618,092	17,530,162	94.2
Pcon14	DD24_b	31,096,297	30,465,329	28,232,290	92.7
Pcon15	DD24_c	21,487,791	21,050,367	19,623,940	93.2
Pcon16	DD36_a	23,668,267	23,190,410	21,286,467	91.8
Pcon17	DD36_b	28,208,438	27,626,313	25,926,388	93.8
Pcon18	DD36_c	24,131,812	23,625,427	22,375,339	94.7

Three independent biological replicates (a-c) were analyzed for each time point. RNA-seq, RNA sequencing.

**Table 2:** RNA-seq results for putative clock-controlled genes (*ccgs*) that were tested by RT-qPCR

Gene	LOX	DESeq	p/padj	Product; Acc. No. of Homolog With Predicted or Described Function
Putative morning-specific *ccgs*				
* PCON_04080*	1.09	0.82	0.00/0.04	Protein of unknown function
* PCON_04507*	1.12	0.94	0.00/0.04	D-galactonate dehydratase; EHA19069.1
* PCON_09334*	1.29	1.11	0.00/0.20	periodic tryptophan protein 2; P25635.2
* PCON_13506*	1.32	1.04	0.00/0.00	subtilisin-like protease; C5PCX1
Putative evening-specific *ccgs*				
* PCON_01669*	−0.70	−0.88	0.00/0.03	mannitol-1-phosphate 5-dehydrogenase; Q9CLY7
* PCON_03866*	−0.60	−0.79	0.00/0.13	trehalose phosphorylase; A6YRN9
* PCON_08647*	−1.25	−1.39	0.00/0.03	UPF0591 membrane protein C15E1.02c; Q9UTI9
* PCON_09693*	−2.19	−2.45	0.00/0.00	fibrinogen alpha chain; P02672
* PCON_12782*	−1.96	−1.76	0.00/0.00	protein of unknown function

Log_2_ values of ratios calculated with LOX or DESeq are given, as well as *P*-values and padj values for DESeq. RNA-seq, RNA sequencing; RT-qPCR, reverse transcription quantitative polymerase chain reaction.

The two novel *ccgs* encode a protein of unknown function (*PCON_04080*), and a WD40-repeat protein (*PCON_09334*) that is a 90S pre-ribosomal component required for proper cell cycle and bud morphogenesis in *Saccharomyces cerevisiae* (Dosil and Bustelo 2004; Shafaatian *et al.* 1996) (Table 2). The two genes that are not strongly rhythmic, but might be under weak clock-control encode a putative D-galactonate dehydratase (*PCON_04507*) that might be involved in sugar acid metabolism (Motter *et al.* 2014), and a putative protease (*PCON_13506*).

It might appear somewhat surprising that *frq* (*PCON_09365*) was not included in the significantly differentially expressed genes in our RNA-seq results (Table S2). One possibility for this might be that only two time points rather than a full time course were chosen for RNA-seq analysis, and therefore slight shifts of peak times might lead to lower signal to noise ratios.

### Some ccgs are also light-regulated

**Figure 4:**
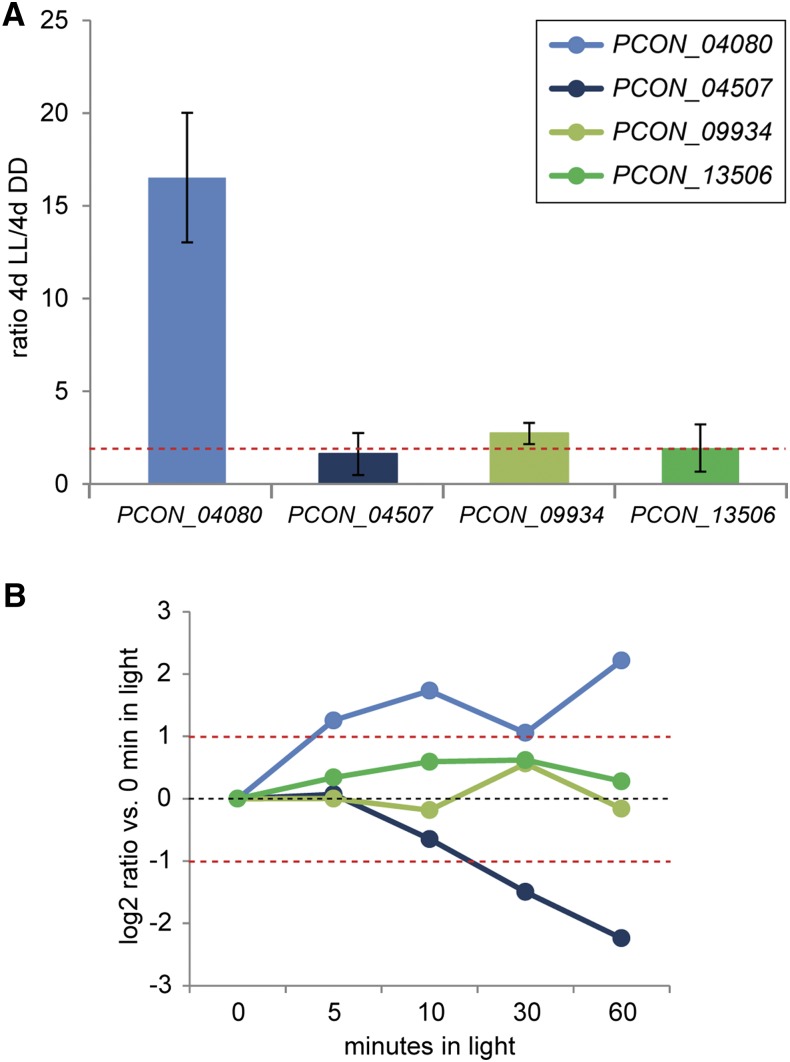
Analysis of light-dependent expression of two putative *ccgs* and two putative weakly clock-controlled genes by RT-qPCR. (A) Gene expression after growth for 4 d in constant light (LL) *vs.* constant darkness (DD). Mean ratios and standard deviations of two independent biological replicates are shown. A red dashed line indicates twofold up-regulation. (B) Gene expression ratios after growth for 4 d in constant darkness and subsequent light induction for 5−60 min. Mean ratios of two independent biological replicates are shown. Standard deviations are left out for clarity, values including standard deviations are given in Table S3. Dashed red lines indicate twofold up- or down-regulation.

In *N. crassa*, light signaling and the circadian clock share several components, and light is one of the factors that can entrain the circadian rhythm (Dunlap *et al.* 2007; Fuller *et al.* 2014). *P. confluens* shows strong reactions to light (including sexual development, which is completely light-dependent), and many genes, including *frq*, are light-induced (Traeger *et al.* 2013). Therefore, we tested whether the novel *ccgs* also might be controlled by light. We analyzed transcript levels of the two *ccgs* after long-term light induction (4 d), as well as induction after short light pulses from 5 to 60 min (Figure 4). The long-term light induction was chosen, because this is a biologically relevant condition under which *P. confluens* is able to develop fruiting bodies, whereas it is sterile in complete darkness. Under long-term light induction, *PCON_04080* is strongly up-regulated, and *PCON_09934* is marginally upregulated (Figure 4A). In the short-term light induction experiments, *PCON_04080* is also up-regulated, and *PCON_09934* is not differentially expressed (Figure 4B and Table S3). Thus, the two *ccgs* do not show a uniform reaction to light, but reveal distinct expression profiles under different light conditions. The two probably weakly clock-controlled genes, *PCON_04507* and *PCON_13506*, are not differentially expressed under long-term light induction, whereas *PCON_04507* is down-regulated in the short-term light induction experiments (Figure 4).

### Analysis of light-entrainment of ccgs

An important property of circadian clocks is the ability to become entrained to external stimuli (“zeitgeber”), *e.g.*, to daily cycles of light and temperature. The result of entrainment is that the period of the rhythm becomes equal to that of the entraining stimuli, and that a stable phase relationship is established between the external stimuli and the entrained rhythm (Johnson *et al.* 2003). In true entrainment, this means that the phase relationship depends on the period length of the zeitgeber and can be tested by using zeitgeber cycles that are shorter or longer than 24 h. If the phase angle of the resulting rhythm is different and specific for different zeitgeber period lengths, this is a good indication of entrainment. However, if the resulting rhythm shows the same phase relationship to the zeitgeber regardless of zeitgeber period lengths, it is likely to be driven, not entrained (Johnson *et al.* 2003; Nsa *et al.* 2015). Thus, for a ∼24-hr, free-running rhythm and a morning-specific gene, in case of light entrainment one would expect transcript levels to rise before the “lights on” with zeitgeber period lengths longer than 24 hr (*e.g.*, in 14-hr DD / 14-hr LL cycles), and after “lights on” with zeitgeber period lengths shorter than 24-hr (*e.g.*, in 9-hr DD / 9-hr LL cycles).

**Figure 5:**
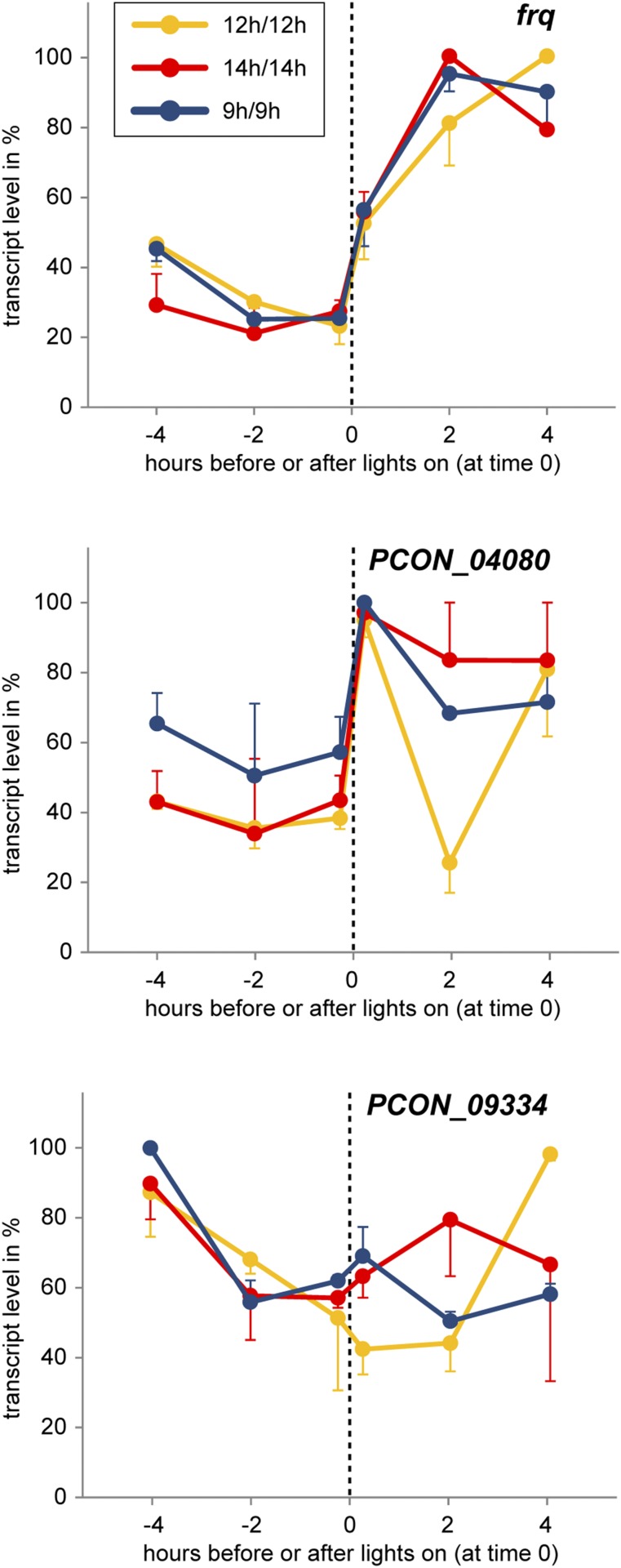
Analysis of light-entrainment for three morning-specific genes by RT-qPCR. Cultures were subjected to dark/light cycles (9 hr/9 hr, 12 hr/12 hr, and 14 hr/14 hr). Samples were harvested at specific time points before or after “lights on” (time 0, dashed vertical line) on day 4 (for entrainment schedule, see Figure S2). Analyses were performed for two independent replicates for each light regime, the greatest value in each experiment was set to 100%, and the mean of two experiments is shown. Standard errors are shown in one direction only (up or down) for better visualization.

We tested whether the putative *ccgs* including *frq* can be light-entrained in *P. confluens*. The putative *ccgs* are morning-specific under free-running conditions, and therefore would be expected to increase expression around the time of “lights on” in dark/light cycles. We therefore analyzed expression at six time points covering a time frame from 4 hr before to 4 hr after “lights on” in three different dark/light regimes (9 hr/9 hr, 12 hr/12 hr, 14 hr/14 hr) (Figure 5). Light induction was observed for *frq* but not consistently for the other *ccgs*. For *PCON_04080*, a short-term increase was observed in transcript levels 0.25 hr after “lights on,” which reflects the light induction that was also observed in the short-term light experiments (Figure 4B). In the case of *frq*, the increase in transcript levels coincides with “lights on” regardless of zeitgeber lengths, which is not consistent with entrainment, but rather with a driven (*i.e.*, synchronized) rhythm. For the putatively weakly rhythmic genes *PCON_04507* and *PCON_13506*, no light entrainment was found either (data not shown). Therefore, at present there is no evidence for light entrainment of *P. confluens* circadian rhythms.

## Discussion

In this study, we have shown that the *frq* gene in the basal filamentous ascomycete *P. confluens* shows two of the three main properties that characterize a circadian rhythm (∼24-hr free-running period length, temperature compensation). Furthermore, we identified two additional *ccgs* by RNA-seq that also show these two properties. Thus, a circadian clock seems to be active in *P. confluens* at the molecular level, even though at present no morphologic output is known. A similar finding was made in *Aspergillus nidulans*, where no developmental rhythms were observed, but expression of the *gpdA* gene was shown to be under circadian control (Greene *et al.* 2003). Entrainment of *gpdA* in *A. nidulans* required the combined input of light and temperature cycles, because neither light nor temperature alone was sufficient for entrainment (Greene *et al.* 2003). A similar phenomenon might be at work in *P. confluens*, where we found that light is able to drive, but not entrain *frq* rhythms (Figure 5).

The surprisingly high conservation of several domains of FRQ in *P. confluens* might lead to the hypothesis that the *P. confluens frq* is part of a circadian oscillator itself. If *frq* were part of the oscillator that would suggest that the FWO is an evolutionary conserved molecular mechanism that was already present in the ancestor of filamentous ascomycetes. However, *frq* might very well have been part of the clock-controlled output first, being recruited into the core oscillator only in selected groups like the Sordariomycetes later during evolution. Our data do not allow conclusions on whether *frq* is part of the oscillator, or part of the output controlled by a circadian clock in *P. confluens*.

One point to note is the high variability in replicate time course experiments for *ccg* expression including *frq* (large error bars in Figure 2). One possible explanation might be that the *P. confluens* circadian rhythm did not evolve to the same level of robustness as found in *N. crassa*, and in the recently described circadian clock in *B. cinerea* (Hevia *et al.* 2015; Hurley *et al.* 2015). The last common ancestor of the lineages leading to *P. confluens* and the other filamentous ascomycetes, respectively, was estimated to have lived 260−413 million years ago (Traeger *et al.* 2013). Therefore, most likely the evolution of circadian rhythms in the early diverging Pezizomycetes *vs.* the other filamentous ascomycetes including *N. crassa* and *B. cinerea* was largely independent. Other possible explanations include the growth conditions or the genetic background in the experiments (see below).

Using RNA-seq, we identified a number of potential *ccgs*. Surprisingly, the number of putative evening-specific genes was much greater among the significantly differentially regulated genes than the number of morning-specific genes (Table S2). However, none of the putative evening-specific genes that were tested could be verified as circadian, whereas two of four morning-specific genes that were tested showed properties of circadian regulation. One reason for this finding might be that the analysis of only two time points is not specific enough to suppress false-positives, and that full time courses are needed (Li *et al.* 2015). In *N. crassa*, the search for *ccgs* started more than 20 years ago, and continues with increasingly sensitive methods (Bell-Pedersen *et al.* 1996; Correa *et al.* 2003; Dong *et al.* 2008; Hurley *et al.* 2014; Loros *et al.* 1989; Nowrousian *et al.* 2003; Zhu *et al.* 2001). In the first analyses by differential cDNA hybridizations, expressed sequence tag sequencing, or microarray analyses, all identified *ccgs* were morning-specific (Bell-Pedersen *et al.* 1996; Loros *et al.* 1989; Nowrousian *et al.* 2003; Zhu *et al.* 2001), with evening-specific genes identified only later (Correa *et al.* 2003; Dong *et al.* 2008). A recent comprehensive screen by RNA-seq identified both evening- and morning-specific genes, but showed that the majority of *N. crassa ccgs* are morning-specific (Hurley *et al.* 2014). The results of our *ccg* verification experiments in *P. confluens* might suggest that the majority of *ccgs* are morning-specific in this fungus, too. However, one has to keep in mind that the growth conditions that are used to assess circadian rhythmicity, namely shaken cultures that can only develop vegetative mycelium, are not natural conditions for this fungus, and that some genes might not be expressed at levels high enough for meaningful quantification, or might show different patterns of regulation compared with a more natural environment. Another reason for the low number of identified *ccgs* and the high standard deviations in repeat experiments analyzing *ccg* expression might be the genetic background in our experiments (*P. confluens* wild type). In *N. crassa*, experiments to analyze circadian rhythms are usually carried out in a *band* mutant that shows amplified circadian output signals compared with the wild type (Belden *et al.* 2007).

In *N. crassa* and other organisms, the circadian clock controls transcript levels of up to 40% of all genes and influences pathways ranging from metabolism to development and mitosis (Bennett *et al.* 2013; Hong *et al.* 2014; Hurley *et al.* 2014). Similar findings for the number and functions of *ccgs* were made in other organisms, *e.g.*, *Arabidopsis thaliana* and mice (15–40% of genes under clock control) (Covington *et al.* 2008; Hughes *et al.* 2009; Li *et al.* 2015). The *P. confluens ccgs* and putative weakly clock-controlled genes that we identified have different predicted functions, suggesting that in *P. confluens*, similar to *N. crassa*, the circadian clock might influence a variety of cellular pathways, and that many more *ccgs* remain to be discovered in this fungus. What biological role a circadian clock in *P. confluens* might play is at present unknown. One possibility is that it might be involved in timing of maturation or ejection of sexual spores. *P. confluens* is a soil-fungus, and in its natural environment, namely forests in temperate climates, fruiting bodies usually appear on the ground after forest fires (Seaver 1909). The spores can be dispersed by wind, and one might hypothesize that the optimal timing of spore ejection could be aided by a circadian clock.

In summary, our data indicate that a circadian clock with a rhythmically expressed *frq* gene is present in the basal filamentous ascomycete *P. confluens*. This suggests that *frq* was already associated with the circadian clock in the last common ancestor of filamentous ascomycetes.

## 
